# Relationship between objective measures of hearing discrimination elicited by non-linguistic stimuli and speech perception in adults

**DOI:** 10.1038/s41598-021-98950-5

**Published:** 2021-10-01

**Authors:** Hugo Sohier, Fabrice Bardy, Teresa Y. C. Ching

**Affiliations:** 1 Faculté de Pharmacie, Université de Montpellier, Montpellier, France; 2 National Acoustic Laboratories, Sydney, NSW Australia; 3 University of Auckland, Auckland, New Zealand; 4 Macquarie University, Sydney, NSW Australia

**Keywords:** Diagnostic markers, Predictive markers, Pathology

## Abstract

Some people using hearing aids have difficulty discriminating between sounds even though the sounds are audible. As such, cochlear implants may provide greater benefits for speech perception. One method to identify people with auditory discrimination deficits is to measure discrimination thresholds using spectral ripple noise (SRN). Previous studies have shown that behavioral discrimination of SRN was associated with speech perception, and behavioral discrimination was also related to cortical responses to acoustic change or ACCs. We hypothesized that cortical ACCs could be directly related to speech perception. In this study, we investigated the relationship between subjective speech perception and objective ACC responses measured using SRNs. We tested 13 normal-hearing and 10 hearing-impaired adults using hearing aids. Our results showed that behavioral SRN discrimination was correlated with speech perception in quiet and in noise. Furthermore, cortical ACC responses to phase changes in the SRN were significantly correlated with speech perception. Audibility was a major predictor of discrimination and speech perception, but direct measures of auditory discrimination could contribute information about a listener’s sensitivity to acoustic cues that underpin speech perception. The findings lend support for potential application of measuring ACC responses to SRNs for identifying people who may benefit from cochlear implants.

## Introduction

People with hearing loss experience difficulties in understanding speech, partly arising from reduced audibility and partly from their reduced ability to discriminate sounds that are audible. Hearing aids amplify sounds so that they become audible to the user but cannot enhance the ability of the user to analyze spectro-temporal variations in the speech wave to discriminate sounds. As hearing loss increases, the ability to perceive the spectral variations decreases^1,2^, thereby resulting in poor speech perception^3^. When hearing aids provide limited benefits to users, they can be considered candidates for cochlear implantation.

Current evaluations of the benefit of hearing aids typically relies on using speech perception tests to measure the accuracy with which words or sentences can be repeated. However, performance in language-based hearing tests relies not only on extraction of acoustic cues of speech, but also semantic (meaning), syntactic (grammar) and other circumstantial (speaker identity, subject matter, listening environment, etc.) cues. When the goal is to assess the listener’s ability to discriminate sounds, it has been proposed that non-linguistic stimuli that are modulated in the frequency domain to mimic the spectral variations found in speech are valuable for this purpose^4–6^.

The non-linguistic stimuli, commonly referred to as spectral ripple noise (SRN), can be varied in terms of number of modulations per octave and depth of modulation^7^. Davies-Venn et al*.* demonstrated that the ability to perceive SRN modulation depth in noise (Spectral Modulation Detection: SMD) was significantly correlated with speech perception in quiet and in noise^4^. In a similar vein, Litvak et al. found a correlation between SMD and consonant and vowel perception^8^. These studies that showed a significant correlation between behavioral discrimination measured using SRN and speech perception assessed using language-based material lend support to the use of SRN to evaluate auditory spectral processing abilities.

However, measurement of behavioral discrimination using SRN cannot be applied to people who are not able to provide behavioral responses reliably, including but not limited to those who may have a language barrier or cognitive limitations or use non-verbal communication modes or too young. Therefore, it is important to examine whether electrophysiological methods for measuring auditory discrimination using the SRN is viable and valid. Won et al*.* reported a significant correlation between behavioral detection of a phase inversion in an SRN stimulus and electrophysiological measure of cortical auditory evoked potential (CAEP) to sound changes or the acoustic change complex (ACC) to the SRN stimulus in three normal-hearing adults^9^. Higher amplitude of the ACC was significantly correlated with better behavioral discrimination ability. The ACC can be reliably recorded with good test–retest reliability not only from listeners with normal hearing but also from those with hearing loss, using hearing aids or cochlear implants (see review by Kim et al., 2015)^10^. As psychoacoustic measures of discrimination were related to speech perception^8,11,12^, and psychoacoustic measures of discrimination had been related to electrophysiological measures of discrimination^13^, it may be surmised that objective ACC measures induced by spectral variation in SRNs are related to subjective speech perception. There is some evidence that ACCs elicited using synthesized vowel tokens were related to behavioural detection in normal-hearing adults^14^; and that the latency of ACCs elicited using changing pure tones were correlated with speech perception scores in adults using cochlear implants^15^. The current study extends previous work by investigating the relationship between ACCs and behavioral discrimination elicited using SRN and speech perception.

It is widely known that CAEPs are influenced by characteristics of the stimulus, including the level of presentation. Cheek et al. showed that synthesized vowel tokens presented at 70 dBA elicited higher ACC amplitude than tokens presented at 40 dBA in normal-hearing listeners^14^. However, this effect may be different in people aided using hearing aids as audibility of the stimulus is determined not only by the signal level relative to the hearing threshold (as in normal-hearers) but also to the hearing aid gain and the internal noise level with amplification^16^. Because hearing sensitivity loss is generally more severe in the high than in the lower frequencies, and spectral processing abilities reduce with increase in hearing loss, it is important to increase knowledge about the effects of high- or low-pass filtering and presentation level of the SRN on the amplitude of ACCs in people using amplification, so that ACC measurements can be used to optimize hearing aids or to assess whether cochlear implants need to be considered to meet individual listening needs.

In this study, we aimed to investigate the relationship between subjective speech perception and objective ACC responses to sound changes measured using SRN. The findings will have significant implications on whether ACC responses to SRN can be used to guide intervention for hearing loss including potential decisions for cochlear implantation. Firstly, we evaluated the relationship between behavioral measures of phase inversion discrimination (or PID) and speech perception in quiet and in noise by adults with normal hearing (NH) and those with hearing loss (HL). In line with current knowledge, we hypothesized that better PID is related to better speech perception in quiet and in noise. Secondly, we examined the relationship between behavioral PID and cortical ACC responses to SRN. We hypothesized that better behavioral discrimination (lower detection thresholds for phase inversion) is related to stronger cortical ACC responses (higher cortical response amplitude). We also hypothesized that ACC response amplitude increases with higher presentation level. Thirdly, we examined the relationship between cortical ACC responses to SRN and speech perception in quiet and in noise. We hypothesized that people who had better speech perception performance also had better ACC performance and better psychoacoustic PID in response to SRN. Performance in all three measures will be affected by the participants’ hearing threshold level.

## Results

### Speech perception

For speech perception in quiet at 50% correct, the mean speech reception threshold (SRT) for normal hearing (NH) listeners was 18.7 dB SPL (SD = 5.7). The mean SRT for hearing impaired (HI) listeners was 56.9 dB SPL (SD = 12.9). For sentence perception in babble noise at 50% correct, the mean SRT in terms of signal-to-noise ratio (SNR) for the NH group was − 0.1 dB (SD = 1.1). The mean SNR for HI listeners was 3.9 dB (SD = 2.3 dB). A 2 × 2 repeated measures ANOVA with SRT (in Quiet and in Noise) as dependent variables and hearing status (NH and HI) as categorical variable showed significant interactions. Whereas NH listeners had better speech perception than HI listeners in quiet, there was no significant difference in performance in noise (*p* < 0.0001).

### Phase inversion discrimination threshold (PID)

Table 1 summarizes the PID thresholds for NH and HI listeners for a low-pass- (LP) and a high-pass-filtered (HP) stimulus. A 2 × 2 repeated measures ANOVA with PID thresholds (HP and LP) as dependent variables and hearing status (NH and HI) as categorical variable showed that on average, NH listeners had better (lower) discrimination thresholds than HI listeners (*p* < 0.05). The main effect of filter condition was also significant – the discrimination thresholds for a low-pass filtered SRN were significantly lower (better) than those obtained for a high-pass filtered SRN (*p* < 0.001). The interaction effect was not significant (*p* > 0.05).

**Table 1:** Descriptive statistics for speech perception (speech reception threshold or SRT) and phase inversion discrimination (PID) for high-pass and low-pass filtered stimuli are shown for normal hearing (NH) and hearing-impaired (HI) subjects.

	n	SRT quiet			SNR noise			PID High Pass			PID Low Pass		
		M	SD	95%CI	M	SD	95%CI	M	SD	95%CI	M	SD	95%CI
NH	13	18.66	5.66	3.08	− 0.04	1.05	0.57	8.53	3.15	1.71	5.83	3.96	2.15
HI	10	56.87	12.92	8.01	3.91	2.29	1.42	16.82	9.02	5.59	10.29	5.66	3.51
Total	23	35.27	21.47	8.77	1.68	2.60	1.06	12.14	7.51	3.07	7.77	5.17	2.11

Mean (M), standard deviation (SD) and 95% confidence intervals (95%CI) are shown.

### Electrophysiological measure of onset detection and ACC discrimination

The cortical P1-N1-P2 complex is a cortical auditory evoked potential elicited by the onset or offset of an acoustic stimulus or changes within an acoustic stimulus^17^. The cortical response to the onset of a stimulus is referred to here as “onset” denoting detection of a sound at the cortical level. The cortical response to a phase change within the stimulus is referred to as the ACC. Table 2 shows the z-scores for onset detection and ACC discrimination, separately for NH and HI participants. A 2 × 2 × 2 repeated measures ANOVA was performed on the z-scores (two response types: onset and ACC; two frequencies: high-pass and low-pass filtered; and two presentation levels: normal and low) with hearing status (NH vs HI) as a categorical factor. The main effects of response type, frequency, and level were significant (*p* < 0.0001, *p* < 0.0001, and *p* < 0.05 respectively). There were significant interactions between response type and hearing status (*p* < 0.01). Post-hoc analyses using Bonferroni comparisons revealed that NH subjects had significantly better ACC scores (lower z-score) than HI subjects, but there was no significant difference in onset scores. There were significant interactions between response type and frequency (*p* < 0.05). On average, ACC responses at high frequencies were poorer (lower in amplitude) than those at lower frequencies. Also, there were significant interactions between response type and level (*p* < 0.05). On average, onset detection was better at normal levels than at lower levels, but discrimination ACC was not significantly different between levels (*p* > 0.05).

**Table 2:** Mean z-scores of cortical responses to onset (top panel) and acoustic change complex (ACC) (bottom panel) for normal-hearing (NH) and hearing-impaired (HI) subjects.

	n	HP20NL			HP20LL			LP20NL			LP20LL		
		M	SD	95%CI	M	SD	95%CI	M	SD	95%CI	M	SD	95%CI
**ONSET**													
NH	13	− 5.84	2.32	1.26	− 5.34	2.16	1.18	− 6.86	2.57	1.39	− 6.39	2.55	1.38
HI	10	− 6.09	1.99	1.23	− 3.91	1.93	1.20	− 6.85	2.17	1.35	− 6.23	2.81	1.74
Total	23	− 5.95	2.13	0.87	− 4.72	2.15	0.88	− 6.85	2.35	0.96	− 6.32	2.60	1.06
**ACC**													
NH	13	− 4.60	1.66	0.90	− 4.23	2.14	1.16	− 6.14	1.85	1.01	− 6.27	1.92	1.04
HI	10	− 2.14	1.77	1.10	− 1.74	1.53	0.95	− 4.53	1.99	1.23	− 4.53	1.68	1.04
Total	23	− 3.53	2.08	0.85	− 3.15	2.24	0.92	− 5.44	2.04	0.83	− 5.51	1.98	0.81

The four stimulus conditions were: HP20NL (high-pass filtered, 20 dB modulation depth, presented at normal conversation level of 65 dB SPL), HL20LL (high-pass filtered, 20 dB modulation depth, low level of 55 dB SPL), LP20NL (low-pass filtered, 20 dB modulation depth, normal level), and LP20LL (low-pass filtered, 20 dB modulation depth, low level). Mean (M), standard deviation (SD), and 95% confidence intervals (95%CI) are shown.

### Correlation analyses

Results of correlations among speech perception, phase inversion discrimination and cortical discrimination and detection are summarized in Table 3. On average, significant correlations were found between speech perception and phase inversion discrimination and cortical ACCs for the 23 participants. There were also significant correlations between speech perception and averaged hearing level (Quiet: *r* = 0.91, *p* < 0.001; Noise: *r* = 0.85, *p* < 0.001).

**Table 3:** Relationships (Product moment correlations) among speech perception, phase inversion discrimination and cortical responses.

Variable		Speech perception		PID		ACC				ONSET				BE4FA
		SRT quiet	SRT noise	High Pass	Low Pass	HP20NL	HP20LL	LP20NL	LP20LL	HP20NL	HP20LL	LP20NL	LP20LL	
Speech Perception	SRT quiet	1.00	0.78 ***	0.62 **	0.45 *	0.62 **	0.57 **	0.53 **	0.51*					0.91 ***
	SRT noise		1.00	0.72 ***	0.59 **	0.70 **	0.60 **	0.44 *	0.51 *					0.85 ***
PID	High Pass			1.00	0.88 ***		0.59 **	0.52 *						0.50 *
	Low Pass				1.00		0.42 *	0.50 *						
ACC	HP20NL					1.00	0.57 **				0.63 **			0.67 ***
	HP20LL						1.00	0.62 **			0.76 ***	0.48 *	0.42 *	0.56 **
	LP20NL							1.00	0.56 **		0.54 **		0.42 *	
	LP20LL								1.00				0.57 **	0.53 *
ONSET	HP20NL									1.00	0.60 **	0.49 *	0.56 **	
	HP20LL										1.00	0.67 ***	0.52 *	0.42 *
	LP20NL											1.00	0.59 **	
	LP20LL												1.00	
BE4FA														1.00

*** indicates *p* < 0.001, ** indicates *p* < .01, and * indicates *p* < 0.05.

**Figure 1:**
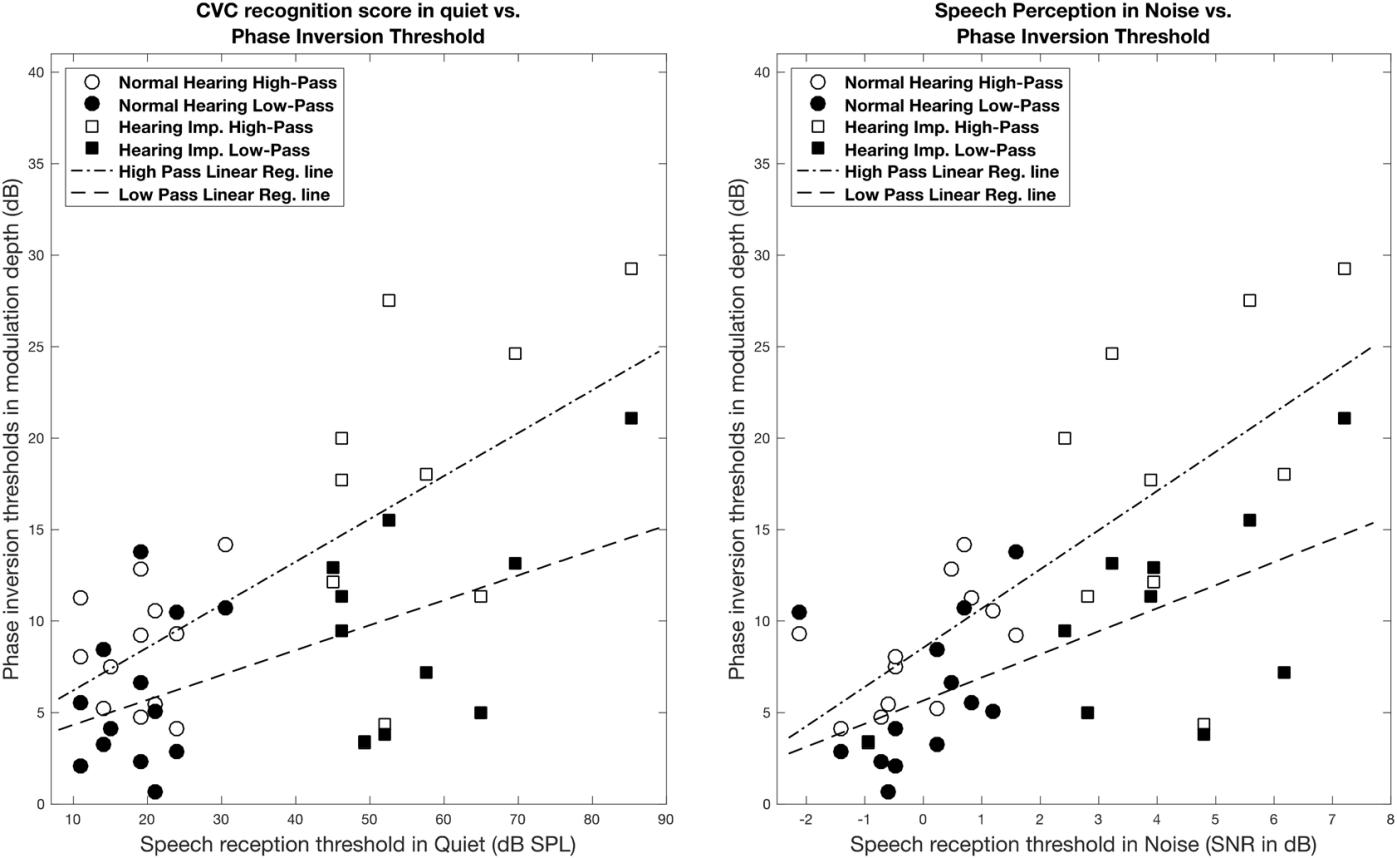
Correlation between the PID thresholds (dB re 100% modulation depth) and speech perception performance. The left panel shows PID as a function of speech reception in quiet. The right panel shows PID as a function of speech perception in noise.

Phase inversion discrimination (PID) thresholds and speech perception scores were correlated. For high-pass filtered stimuli, correlations with speech perception in quiet and noise were significant (Quiet: *r* = 0.62, *p* < 0.01; Noise: *r* = 0.72, *p* < 0.001). Similarly, PID for low-pass filtered stimuli were significantly correlated with speech perception (Quiet: *r* = 0.46, *p* < 0.05; Noise: *r* = 0.59, *p* < 0.01). As shown in Fig. 1, better behavioral discrimination (lower PID expressed as lower modulation depth in dB) measured using high-pass and low-pass filtered SRNs were associated with better speech perception in quiet and in noise (lower speech reception threshold), for adults with normal hearing and those with hearing impairment. Linear regression showed that speech perception in noise SRT was significantly associated with phase inversion discrimination, after controlling for better-ear hearing level (High-pass: *beta* = 0.11, *p* < 0.01; Low-pass: *beta* = 0.10, *p* < 0.01).

**Figure 2:**
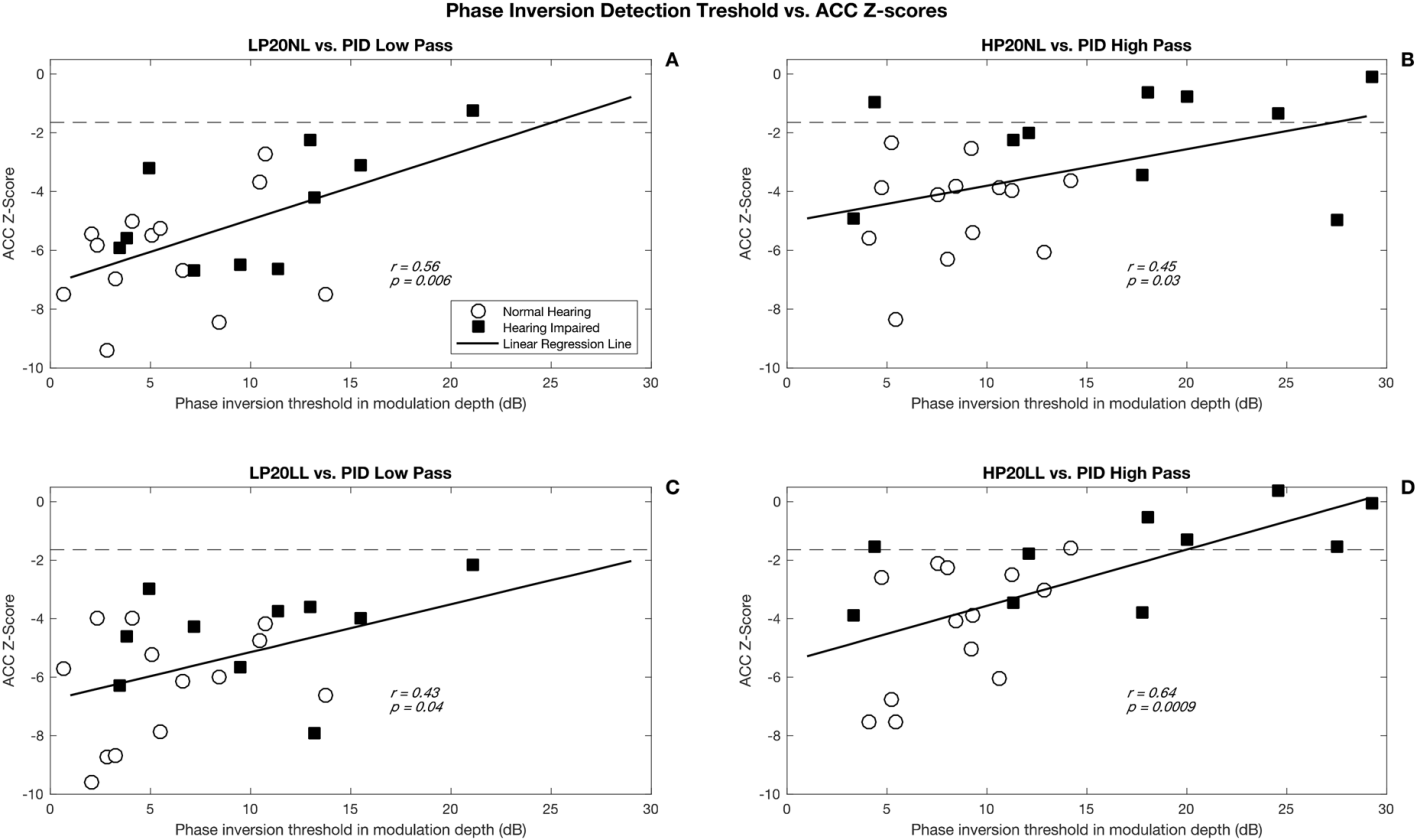
Correlation between PID thresholds (dB) and ACC z-scores for 4 stimuli. Panel (**A**) shows results for the low-pass filtered stimuli presented at normal conversational level (LP20NL), panel (**B**) shows results for the high-pass filtered stimuli presented at normal conversational level (HP20NL), panel (**C**) shows results for the low-pass filtered stimuli presented at low level (LP20LL), and panel (**D**) shows results for the high-pass filtered stimuli presented at low level (HP20LL).

Phase inversion discrimination was significantly correlated with cortical ACCs (*p* < 0.01) but not cortical onset responses (*p* > 0.05). As shown in Fig. 2, better behavioral discrimination (lower PID) was associated with stronger cortical ACC responses (lower z-scores depicting higher cortical response amplitude).

**Figure 3:**
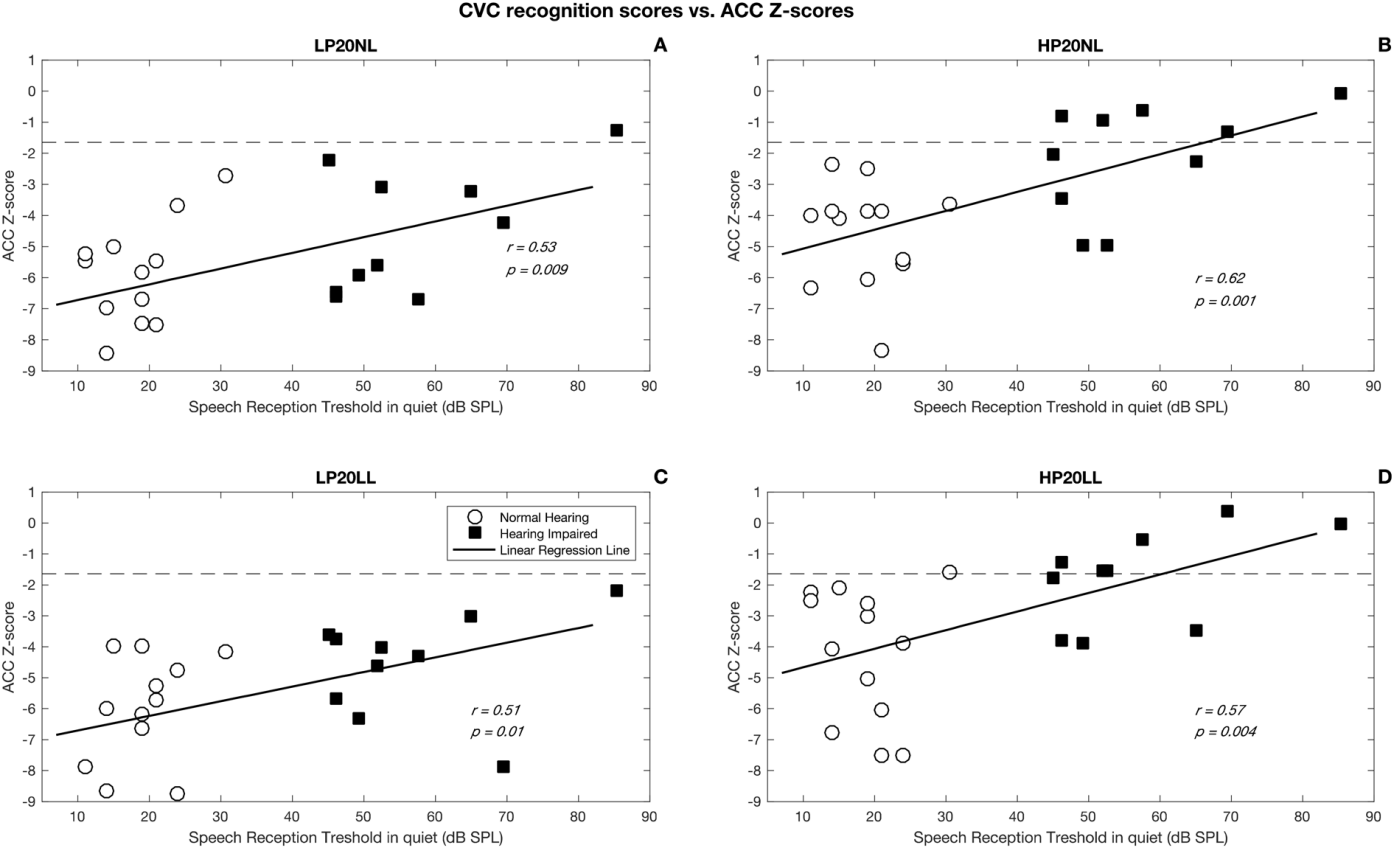
Correlation between speech perception in quiet and ACC z-scores for 4 stimuli. Panel (**A**) shows results for the low-pass filtered stimuli presented at normal conversational level (LP20NL), panel (**B**) shows results for the high-pass filtered stimuli presented at normal conversational level (HP20NL), panel (**C**) shows results for the low-pass filtered stimuli presented at low level (LP20LL), and panel (**D**) shows results for the high-pass filtered stimuli presented at low level (HP20LL).

**Figure 4:**
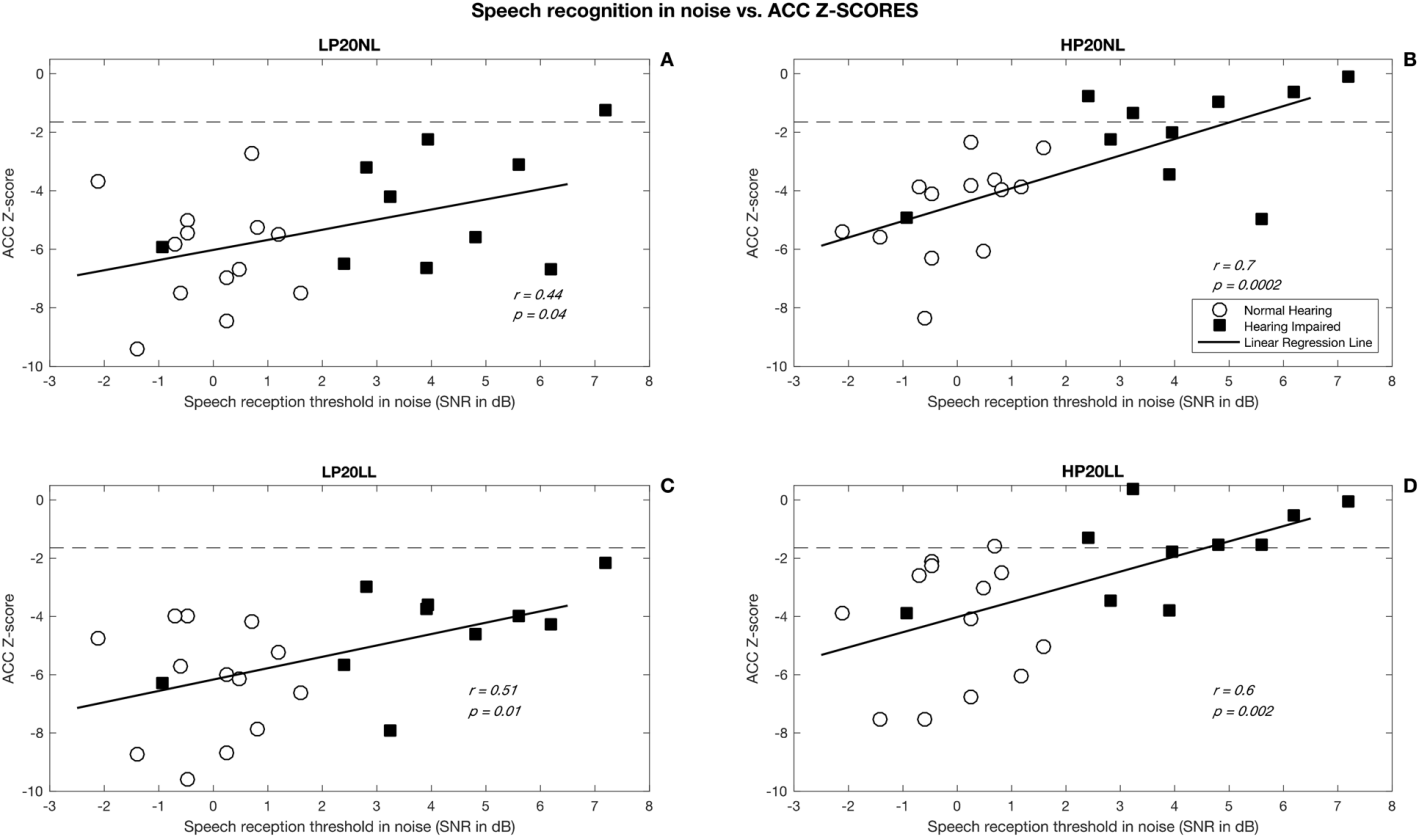
Correlation between speech perception in noise and ACC z-scores for 4 stimuli. Panel (**A**) shows results for the low-pass filtered stimuli presented at normal conversational level (LP20NL), panel (**B**) shows results for the high-pass filtered stimuli presented at normal conversational level (HP20NL), panel (**C**) shows results for the low-pass filtered stimuli presented at low level (LP20LL), and panel (**D**) shows results for the high-pass filtered stimuli presented at low level (HP20LL).

Speech perception was significantly correlated with cortical ACC (*p* < 0.05) but not onset responses (*p* > 0.05). As shown in Figs. 3 and 4, subjects with cortical ACCs that were higher in amplitude were better at perceiving speech in quiet and in noise. The strongest correlation (*r* = 0.70, *p* < 0.0001) was found between speech perception in noise and ACCs measured with high-pass filtered SRNs presented at normal conversational level (HP20NL). Linear regression analysis revealed that this association between speech perception and ACC z-score was no longer significant (*p* > 0.05) after controlling for the effect of audibility. It is possible that there are associations, but the current sample size was not large enough to detect them.

## Discussion

The current study investigated the relationship between subjective speech perception and objective measures of auditory cortical responses induced by phase changes in SRN. A significant relationship would suggest a potential applicability of using objective measures of ACC to SRN as a predictor of hearing aid benefit for speech perception. This method may be used to identify patients who may benefit from cochlear implantation, especially those who cannot perform reliably in behavioral speech perception testing.

This study found a significant correlation between behavioral SRN modulation depth threshold and sentence perception in quiet and in noise in NH and HI listeners (Fig. 1). Better phase inversion discrimination was associated with better speech perception. This association was significant after controlling for the effect of hearing level (*p* < 0.01). Our results obtained using a phase inversion paradigm are consistent with previous research that used a SMD task to find modulation depth thresholds. Davies et al. and Won et al. demonstrated that the subjective ability to perceive SRN modulation depth and discrimination were significantly correlated with word recognition in quiet and in noise^4,9^. This lends support to applying SRN stimuli in electrophysiological measurements of spectral discrimination ability.

Secondly, this study found a significant relationship between behavioral discrimination and cortical ACC responses elicited by SRN. Better behavioral discrimination measured using high-pass and low-pass filtered stimuli was significantly associated with higher ACC response amplitude, but not with onset response. Consistent with previous studies, our results confirmed that ACC can be elicited by a change of spectral characteristics alone in non-linguistic stimuli presented at normal conversational level of around 65 dB SPL and at a low input level of 55 dB SPL^4,9,13,15^. On average, onset response amplitude was higher at higher presentation level, but the amplitude of ACC response was not significantly different between levels. This may be partly related to the extent to which cortical auditory neurons are sensitive to the direction of change of acoustic parameters^18,19^. On average, there was no significant difference in onset detection between NH and HI groups implying that the stimuli were audible and detected by both groups efficiently. However, the cortical ACC responses induced by SRN phase inversion was significantly stronger for NH than for HI people suggesting that HI participants had difficulty discriminating phase changes within the acoustic stimuli. As hearing aids increase audibility but cannot improve discrimination, this electrophysiological test using filtered SRN can be used to identify patients using hearing aids who can hear sounds but cannot discriminate between sounds especially in the high frequencies for cochlear implant candidacy evaluation. Our findings complement those reported by Choi et al. (2016) on using SRNs with spectro-temporal modulations for behavioral testing and extends the application of SRNs for electrophysiological testing^12^.

Thirdly, we found that ACC z-scores were significantly correlated with PID thresholds for high-pass and low-pass filtered SRN in NH and HI adults. These findings complement those reported by Won et al. (2011) that showed a significant relationship between ACC and spectral ripple discrimination^9^. Higher amplitude in ACC (lower z-score) was associated with better thresholds in phase discrimination (lower modulation depth). These results show that the ACC response to phase changes in the SRN could be used as an objective index of the listener’s capacity for extracting acoustic cues that are important in differentiating speech sounds^13,20^.

This study shows that cortical ACC induced by SRN phase inversion correlated significantly with speech perception in quiet and in noise (*p* < 0.001). Higher ACC amplitude was associated with better speech perception ability. This finding is consistent with the report of He et al. (2014) that showed correlations between ACCs evoked by changes in the stimulating electrode in cochlear implants of children with auditory neuropathy and their word scores^21^. We found that speech perception in noise was significantly correlated with ACC z-score for high-pass filtered SRN with a 20 dB modulation depth presented at 65 dB SPL in the free field (*r* = 0.7, *p* < 0.001).

However, when we partialled out the effect of audibility from the speech perception scores and looked at the relationship between speech perception and ACC z-score, there was no longer a significant relationship between speech perception in noise and ACCs for the SRN (*p* > 0.05). This is unlike the finding of a reduced but significant relationship between behavioral phase discrimination and speech perception in noise after controlling for the effect of audibility. Taken together, the findings suggest that audibility is a strong predictor of behavioral and electrophysiological measures of phase discrimination as well as speech perception. This lends support to the current adoption of audiometric criteria for referrals for cochlear implant candidacy in many countries^22^. Nevertheless, previous studies have failed to show a correlation between pre-operative hearing threshold levels and post-operative speech perception outcomes^23–26^. Considerable variability in post-operative outcomes exists. Some evidence suggests that variability may be explained partly by the auditory sensitivity of a user of cochlear implants to acoustic cues underlying speech perception and their attention to those cues^27^. Measurement of a listener’s auditory ability for sound discrimination pre-operatively may contribute prognostic information on likely outcomes. This may be especially useful in cases where audiometric thresholds do not meet candidacy criteria according to local regulatory authorities. Importantly, cortical measurements would assist with identifying cochlear implant candidates in patients diagnosed with auditory neuropathy spectrum disorder ^28^. The use of SRNs for assessing auditory discrimination has the potential for adoption across regions of diverse languages. The viability and validity of using electrophysiological measures of ACC to assess auditory discrimination ability using the SRNs suggest that objective methods can be applied to patients who cannot provide reliable behavioral responses.

Future studies will extend the current work to a larger sample to adequately control for the effect of audibility in electrophysiological measurement of ACC using SRN. It would be useful to perform further research examining in detail how to optimize SRNs for electrophysiological measurements for clinical applications. Further work will also extend ACC assessments to infants and young children for determining detection and discrimination of sounds with hearing aids. In infants with congenital hearing loss, providing early implantation is crucial to maximizing outcomes^29^.

## Conclusion

The current study showed a significant correlation between speech perception and spectral discrimination abilities measured using psychoacoustic methods and electrophysiological methods, with SRN as stimuli in adults with normal hearing or with hearing loss using hearing aids. Cortical ACC induced by a phase inversion in an SRN was strongly correlated with speech perception in quiet and in noise. Cortical ACC was also significantly correlated with phase inversion discrimination measured using psychoacoustic methods. The ACC responses elicited using non-linguistic stimuli have the potential to be used as a clinical tool to objectively predict speech abilities for people who cannot provide reliable behavioral responses in speech perception testing. This objective marker can become a useful criterion for optimizing hearing aids and for identifying candidates for cochlear implantation.

## Methods

### Subjects

Twenty-three adult listeners who were native speakers of English participated in this study, comprising 13 with normal hearing (NH) and 10 with hearing impairment (HI). The NH group comprised 10 females and 3 males aged between 19 and 55 years (mean = 27, SD = 9.6) who had hearing thresholds within the normal range in both ears (averaged hearing thresholds at 0.5, 1, 2 and 4 kHz or 4FA < 20 dB HL). The HI group comprised 3 females and 10 males aged between 36 and 81 years (mean = 68.1, SD = 15.0). Their hearing loss ranged from moderate to profound degrees. All HI participants had worn hearing aids for at least 6 months. The study protocol was approved by the Hearing Australia Human Research Ethics committee. All participants provided written informed consent to participate in this study. This study was carried out in accordance with approved guidelines.

### Equipment for behavioral testing

All the behavioral tests were conducted in free field in an acoustically treated booth. The stimuli were presented from a loudspeaker (B&W 600 Series) placed 180 cm from the subject, via a sound card (Fireface 800) controlled by a MATLAB (The Mathworks, Natick) graphical user interface running on a computer. All stimuli for the behavioral discrimination test were equalized in level in terms of loudness using a loudness model ^30^ implemented in Matlab (The Mathworks, Natick). A half-inch Brüel & Kjaer microphone was used for calibration. Stimuli were presented at an overall level of 20 sones, measured at the subject position with the subject absent. Speech perception tests were calibrated using a Matlab user interface and a Brüel & Kjaer Hand-Held Analyzer type 2250. The overall presentation level of speech stimuli was adjusted to be the same as an equivalent continuous noise (Leq) at 75 dB SPL.

### Audiological assessment

Otoscopy was performed before pure tone audiometry was performed using insert earphones ER3 and an Interacoustic AD 28 Diagnostic Audiometer^31^. Hearing thresholds were measured at octave frequencies between 0.25 and 8.0 kHz in each ear using standard procedures^32^. The four-frequency average (4FA) hearing loss was calculated by computing the mean of the thresholds obtained at 0.5, 1.0, 2.0, and 4.0 kHz in each ear. The hearing aids used by HI participants at their personal settings were measured using an HA2-2cc coupler and a broad-band speech-weighted stimulus using the Aurical Hearing Instrument Test (HIT) box and the OTOsuite software (Natus Medical Inc., Taastrup, Denmark). Average real-ear-to-coupler differences were used to estimate levels of an amplified signal in the real-ear^33^. The real-ear levels were used in calculations of audibility.

### Speech perception

Two different types of speech stimuli were used. Consonant–vowel-consonant (CVC) words were presented in a carrier sentence in quiet, and the Bamford-Kowal-Bench (BKB)-like sentence test material^34^ were presented in babble noise. Training was provided before testing.

#### Speech perception in quiet

Recorded stimuli were presented at 65 dB SPL. Each carrier sentence contained two meaningless words; e.g. I saw the [CVC] in the [CVC]; spoken by a female native speaker of Australian English. Subjects were required to repeat the words they heard. The input level was adjusted in 5 dB steps for the first two reversals, after which the step size was reduced to 2 db. The speech reception threshold or SRT at which 50% of the words were correctly repeated was calculated, based on the average of the last 10 reversals. The test stopped when at least 24 stimuli were presented, and when 10 reversals were obtained with the 2-dB step size with a variance of less than 1 dB. The SRT, expressed in dB SPL, was calculated as the mean of the last 10 reversals.

#### Speech perception in noise

Recorded BKB-like sentences spoken by a male native speaker of Australian English were presented in a babble noise generated using two collocated talkers produced by native Australian English speakers. Subjects were sked to repeat the sentences. The SRT for 50% of the sentences correctly repeated was measured. The target speech was fixed at 65 dB SPL, and the babble noise was adjusted adaptively. The test started at 0 dB SNR, and adjustments were in 5 dB steps for the first two reversals, then in 2-dB steps. The test stopped when the variance was smaller than 1 dB. The SRT, expressed in dB SNR, was calculated as the mean of the last 10 reversals.

### Behavioral discrimination test

Phase inversion discrimination (PID) thresholds were measured using spectrally modulated non-linguistic stimuli (SRN). The modulation depth thresholds for which a 180° phase inversion was perceived 50% of the time was measured.

All the stimuli were created by superposition of 4000 pure tone sounds equally distributed on a logarithmic scale. Two bandwidth conditions were included: a low-pass (LP) filtered SRN with a frequency bandwidth ranging from 250 to 1,500 Hz, and a high-pass (HP) filtered SRN with a frequency bandwidth ranging from 2,000 to 11,200 Hz. The spectral modulation starting phase for the ripple was randomly selected from a uniform distribution (0 to π rad). The ripple density was one ripple per octave. The stimuli had a total duration of 500 ms with a 10 ms up and down ramp. The inter-stimulus-interval (ISI) was 500 ms. To measure the PID threshold, a 3-Alternative-Forced-Choice (3AFC) paradigm, with a two-down one-up procedure was used. During the test, one interval had a 180° phase shift (i.e. deviant signal) compared to two other stimuli (i.e. standard signal). The participant had to select the one that was different from the other two. The position of the deviant stimulus in each triplet was randomized across trials. The modulation depth was initially set to 20 dB for the NH group and 25 dB for the HI group. The modulation depth varied automatically in steps of 2 dB for the first two reversals and 0.5 dB for the last ten. The threshold was defined as the average modulation depth of the last 10 reversals. The overall presentation level was 20 sones. All testing was preceded by training.

### Electrophysiological tests

**Figure 5:**
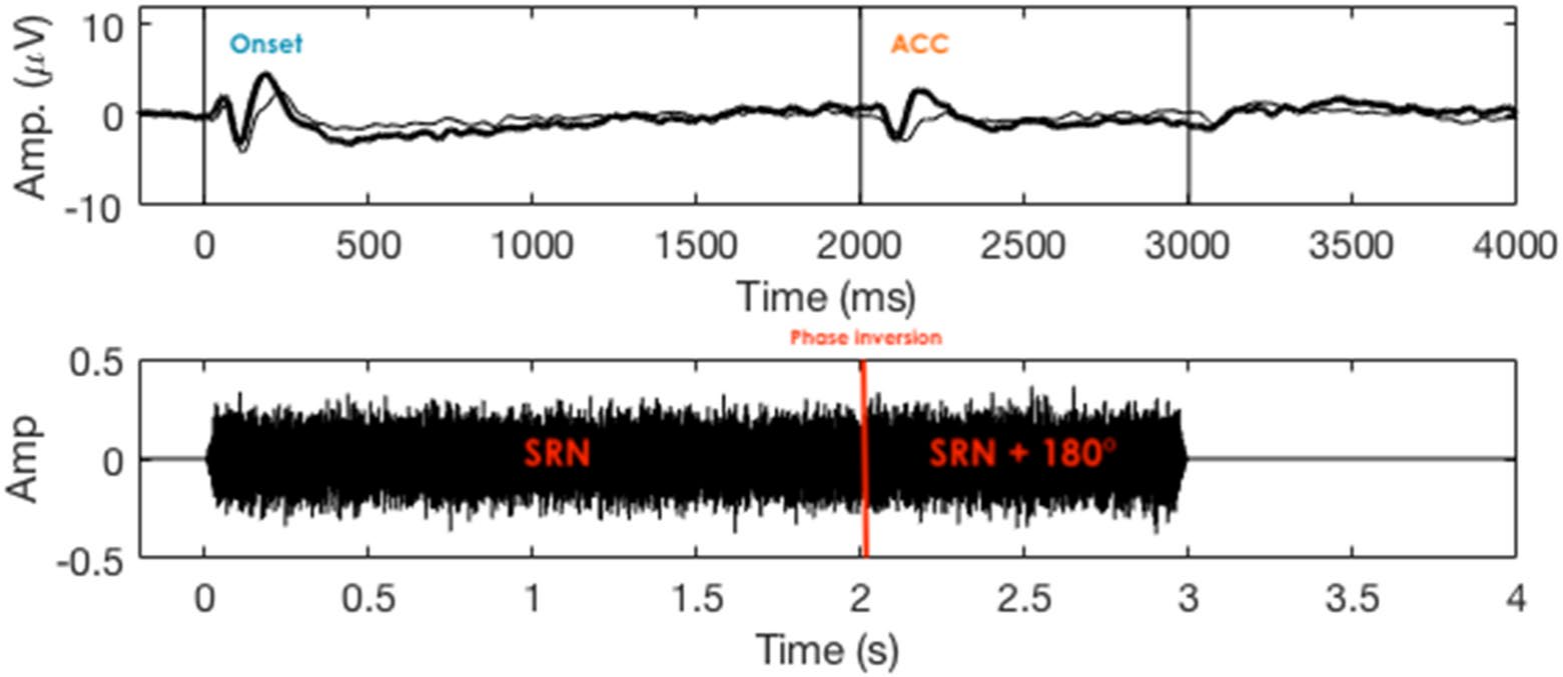
The lower panel shows the waveform of the high-pass filtered spectral ripple noise, bandwidth from 2 kHz to 11.2 kHz, with a phase inversion of 180°, one ripple per octave. The top panel shows the mean EEG recording to the stimulus presented at 20 sones. The cortical responses to the onset and the acoustic change complex (ACC) are shown.

The electrophysiological test consisted of the recording of the electroencephalogram (EEG) in response to SRN stimuli with similar characteristics to the one used for behavioral testing. Electrophysiological assessment consisted of recording the CAEPs elicited by the onset of the SRN and the acoustic change complex (ACC) elicited by a transition between two SRN with phase inversion from 0° to 180° (see Fig. 5). The HEARLab system (Frye Electronics, Tigard, OR, USA) was used to record the raw EEG data.

#### Stimuli

All the stimuli were created by concatenating two SRNs that have a 180° phase inversion, at one ripple per octave. The total duration of the stimulus was 3 s with an SRN transition at 2 s after onset. To avoid spectral splatter at the transition, each stimulus was windowed with a 40 ms rise-fall ramp and the stimuli were concatenated with a 20 ms overlap. Two different modulation depths were investigated: 20 and 50 dB for the high-pass filtered condition. A pilot comparison of responses to high-pass filtered stimuli that have either 20 dB modulation depth or 50 dB modulation depth revealed no significant difference in onset or ACC responses. Therefore, 20 dB modulation depth was used for subsequent testing. The HP and LP stimuli used for PID threshold assessments were used. These were presented at normal conversational level (NL) of 20 sones (or around 65 dB SPL) and at a lower input level (LL) of 55 dB SPL. Four stimuli were used: HP20NL (high-pass filtered, 20 dB modulation depth, presented at normal level); HP20LL (high-pass filtered, 20 dB modulation depth, presented at low level); LP20NL (low-pass filtered, 20 dB modulation depth, normal level); and LP20LL (low-pass filtered, 20 dB modulation depth, low level).

#### Electrophysiological procedure

Testing was conducted in the free field using a single loudspeaker, positioned 100 cm from the subject, at 0° azimuth. All recordings were obtained in a sound-treated booth while the subject sat in a comfortable chair attending to a muted, close-captioned movie of their choice. EEG recording was conducted using four electrodes: one reference electrode placed at FCz and two active electrodes (“M1” on left mastoid and “M2” on right mastoid). The ground electrode was placed on the forehead^35^. For each stimulus, 120 epochs were recorded. The presentation order of stimuli was randomized across participants using a Latin square design^36^. Each condition lasted 13 min for a total of an hour and fifteen minutes approximately.

The EEG data were post-processed using a custom-designed Matlab script. Before analog-to-digital conversion, the signal was high-pass filtered at 0.33 Hz by an analog first-order filter. The signal was down-sampled to 1 kHz and low-pass filtered at 30 Hz with a 128-order zero-time delay filter. The raw EEG of each epoch was cut into two segments corresponding to the onset (CAEP induced during the transition from silence to sound stimulation) and the ACC (CAEP induced by the phase inversion of the SRN). The recording window for each response consisted of a 200 ms pre-stimulus interval and 700 ms post-stimulus interval. An artifact rejection algorithm was then applied to exclude any epoch that had a maximum absolute value threshold greater than 160 μV or a mean epoch value greater than 60 μV. A weighted average of the two channels was calculated for each epoch. Then, each recorded epoch was reduced to voltage levels in nine bins, covering the range from 51 to 348 ms, with each bin being 33 ms wide^37^. The Hotelling’s *T*^*2*^ statistics was used to obtain an objective measure of response detection—a statistically significant *p*-value means that an electrical activity corresponding to a CAEP was detected to be significantly different from zero or background noise. The z-score was computed from the *p*-value of the Hotelling’s *T*^*2*^ for both the onset and the ACC responses for each stimulus condition.

#### Calculation of audibility of stimuli

Audibility was the difference between the signal level and the maximum of hearing threshold and noise level. In HI listeners, real-ear measurements were used to calculate the level of the amplified signal at the ear-drum in the better ear. Audibility was estimated as the maximum value across different bands of one-third octave spectrum on the basis that audibility will most strongly be determined by the frequency region in which it is greatest.

### Data analysis

To determine the influence of hearing status (NH or HI) on performance in speech perception, PID, and cortical onset and ACC responses, analysis of variance (ANOVA) with repeated measures was used. To determine the influence of presentation level (normal and low), and frequency (HP or LP) on cortical onset and ACC responses, ANOVA with repeated measures was used. To examine the relationship between PID and cortical responses, and between PID and speech perception, and between cortical responses and speech perception, Pearson’s product moment correlation analyses were carried out. Linear regression analyses using the SRT in noise as the dependent variable, and better-ear audibility together with either behavioral discrimination or ACC-z scores as predictor variables were conducted to estimate the effect of audibility on the relationship between discrimination and speech perception.
